# Public Perceptions on Non-Pharmaceutical Interventions for West Nile Virus Infections: A Survey from an Endemic Area in Northern Italy

**DOI:** 10.3390/tropicalmed6030116

**Published:** 2021-06-29

**Authors:** Matteo Riccò, Simona Peruzzi, Federica Balzarini

**Affiliations:** 1 Servizio di Prevenzione e Sicurezza Negli Ambienti di Lavoro (SPSAL), AUSL-IRCCS di Reggio Emilia, Via Amendola n.2, I-42122 Reggio Emilia, RE, Italy; 2 Laboratorio Analisi Chimico Cliniche e Microbiologiche, Ospedale Civile di Guastalla, AUSL-IRCCS di Reggio Emilia, I-42016 Guastalla, RE, Italy; simona.peruzzi@ausl.re.it; 3 Dipartimento per la Programmazione, Accreditamento, Acquisto delle Prestazioni Sanitarie e Sociosanitarie (P.A.A.P.S.S.), Servizio Autorizzazione e Accreditamento, Agenzia di Tutela della Salute (ATS) di Bergamo, Via Galliccioli, 4, I-24121 Bergamo, BG, Italy; federica.balzarini@ats-bg.it

**Keywords:** West Nile Virus, knowledge, risk perception, West Nile Fever

## Abstract

During the last decade, cases of West Nile Virus (WNV) have occurred in the Emilia Romagna Region (ERR). Even though the notification rates remain relatively low, ranging from 0.06 to 1.83 cases/100,000 inhabitants, the persistent pathogen’s circulation in settings characterized by favorable environmental characteristics suggests that WNV is becoming endemic to the Po River Valley. This study assesses knowledge, attitudes, and preventive practices toward WNV prevention among residents from 10 high-risk municipalities from the provinces of Parma and Reggio Emilia (total population: 82,317 inhabitants, census 2020). A web-based survey, based on the health belief model, was performed during the month of January 2021, with a convenience sampling of 469 participants from a series of closed discussion groups on social media (i.e., 2.1% of the potential responders). A total of 243 participants knew the meaning of WNV: Of them, 61.3% were aware of previous WNV infections in ERR, 76.5% acknowledged WNV infection as a severe one, but only 31.3% expressed any worry about WNV. Our results irregularly report preventive practices, either collective (e.g., draining standing water from items and the environment, 50.7%; spraying pesticides around the home, 33.0%) or individual (e.g., use of skin repellants when going outdoors, 42.6%). In a multivariate analysis, performed through binary logistic regression, participants reporting any worry towards WNV were more likely to characterize WNV as a severe disease (adjusted odds ratio [aOR] = 20.288, 95% confidence interval [CI] = 5.083–80.972). On the contrary, respondents supporting community mosquito control programs were more likely among people working with animals/livestock (aOR = 13.948, 95%CI = 2.793–69.653), and supporting tax exemptions for mosquito control programs (aOR = 4.069, 95%CI 2.098–7.893). In conclusion, our results suggest that future interventions promoting WNV prevention among residents in ERR should focus on perceptions of vulnerability to WNV, emphasizing the benefits of personal protective behaviors.

## 1. Introduction

West Nile Virus (WNV), the agent of the West Nile Fever (WNF), is an arthropod-borne RNA virus belonging to the genus Flavivirus (family *Flaviviridae*). The usual WNV life cycle requires reservoirs where the pathogen can actively replicate, a competent vector, and final or incidental hosts [1]. In Europe, the main and final hosts of WNV are migrant birds, which harbor the virus without signs of clinical disease [1], and the competent vector is represented by mosquitoes belonging to the genus *Culex* (mainly *C. pipiens*, *C. peregrinus*, and *C. modestus*) and *Aedes*, which acquire and spread the infection at feeding, also supporting viral replication (i.e., sylvatic cycle) [2,3,4]. Larger mammalians (e.g., horses), and particularly humans, usually represent incidental and dead-end hosts, as they can develop invasive infections, but are far less effective in guaranteeing that the high viremia is infectious to mosquitoes [3,5]. Similar to other arboviruses (e.g., Tick-Borne Encephalitis Virus or TBEV, Usutu virus, and Toscana Virus or TosV) [6,7], WNV is actively circulating in European countries [2,3,4,8,9,10,11], that have become endemic in various areas of Eastern, Western, and Southern Europe, with a seasonal trend from April to November, i.e., when competent vector is active. In Italian territory, a significantly higher abundance of the vector of WNV was recorded in warmer and less rainy conditions. These conditions cause virus spillover outside the sylvatic cycle, to humans and/or equids. Most of the human and equine cases in Italy occurred in the northern part of the country, where the ecological conditions are most suitable for WNV transmission. Among the Northern regions, the Po River Valley has been severely affected since the beginning of the last decade [9,12,13,14].

Po River Valley is a very fertile, and well-irrigated territory, particularly between eastern Lombardy and western Emilia Romagna, and represents the most economically developed and densely populated area of the Italian peninsula. Nevertheless, the Po River Valley is characterized by natural waterlogging, predominantly on the right side of the titular Po River. Consequently, circulation of mosquito-borne pathogens has historically found favorable ground in these areas: For instance, malaria has been endemic in the Po River Valley until the 20th century, when the last marsh areas were eventually reclaimed [15], and in 2007 Emilia Romagna hosted the first European outbreak of autochthonous cases of chikungunya [16,17,18]. Not coincidentally, according to a recent review [15], the greater share of Italian cases of neuro-invasive WNV infections (or WNND) between 2012 and 2020 have occurred in Emilia Romagna (36.1%), followed by Veneto (25.1%), Lombardy (21.8%), and Piemonte (9.7%) (i.e., Italian regions belonging to the Po River Valley), with the highest crude incidence rates (0.441 cases/100,000 people, 95%CI 0.378 to 0.511 compared to a national estimate of 0.228 cases/100,000 people, 0.209 to 0.250) [14]. As the majority of WNV infections usually occur asymptomatic or only mildly symptomatic [19,20], WNND cases often represent a reliable proxy for their epidemiological assessment [15,21,22,23]. In other words, it is reasonable that also the greater share of WNV infections did occur in Emilia Romagna Region between 2012 and 2020.

Since 2008 a vaccine constituted by inactivated WNV strain, VM-2, was licensed for use in horses in the EU by the European Medicines Agency (EMA). In this regard, a study carried out in Southern Italy concluded that vaccines used do not alter the overall hemogram picture and health status of horses though it is associated with modulation of humoral immunity, leukocyte populations, and inflammatory markers [24]. Contrariwise, to date, no vaccine is available for human [25,26,27,28]: Even though several vaccines targeting flaviviruses (e.g., TBE, yellow fever, Dengue, Japanese encephalitis, and Kyasanur forest disease) have been made available, none has been licensed for the use against WNV infection [29,30,31]. Moreover, none of the vaccine candidates has progressed further than to phase I/II clinical trial [32]. With treatment options currently limited to the symptomatic care, non-pharmaceutical interventions (NPIS), including personal behaviors aimed to prevent the exposure to disease-carrying mosquitoes (e.g., insect repellent use, and avoiding mosquito bite), and integrated mosquito management, remain therefore essential to cope with WNV [33]. Unfortunately, public compliance with NPIS is limited [34]. Consequently, appropriate identification and investigation of factors that influence and promote both personal protective behaviors (PPB) and community-level interventions can actively contribute to prevention and control programs [28,35,36,37,38].

To investigate the determinants of engaging and promoting NPIS against WNV in the high-incidence area of Emilia Romagna Region, we surveyed the residents of ten municipalities from the Provinces of Parma and Reggio Emilia about the following endpoints: Their awareness of the WNV/WNF, their level of self-perceived risk for WNV infection, the acceptance of NPIS to reduce mosquito sources and exposures. Eventually, as no similar research on the attitudes of potential recipients of WNV vaccine in the European Union is to date available [33], we preventively assessed the acceptance of hypothetical WNV vaccines.

## 2. Materials and Methods

### 2.1. Study Design

**Figure A1:**
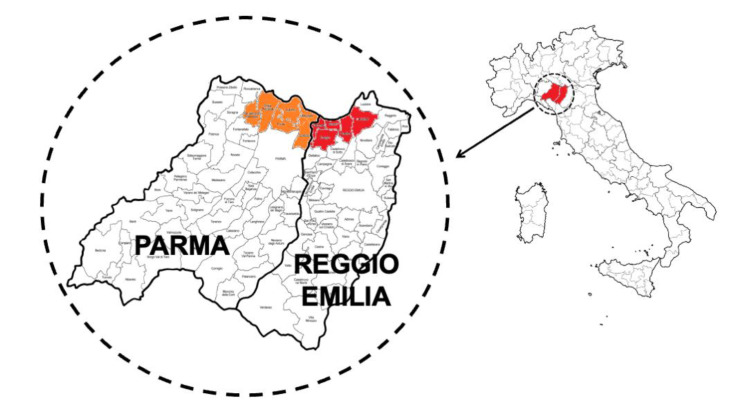
Geographical areas involved in the present survey, i.e., the northern communities of the provinces of Parma and Reggio Emilia, belonging to the Po River basin.

A cross-sectional questionnaire-based study was performed between 01 February 2021 and 10 February 2021, targeting a series of Facebook discussion groups from 10 municipalities of the provinces of Parma and Reggio Emilia from the Po River valley (i.e., San Secondo Parmense, Sissa Trecasali, Colorno, Torrile, Sorbolo Mezzani, Brescello, Poviglio, Boretto, Gualtieri, Guastalla; see Appendix A Table A1 for details), with an overall population of 82,317 inhabitants (census 2020), and a total surface of 437.25 km^2^. The group pages had approximately 22,085 unique members, equals to 26.8% of the target population, with a potentially uneven sampling (range: 10.4–60.1%; Appendix A Figure A1), but no information could be obtained regarding cross-inscriptions, not even how many of these members were actively using Facebook. As no previous studies on KAP towards WNV/WNF have been previously performed in the general population of Emilia Romagna Region, but Health Authorities have promoted several information campaigns, we conveniently assumed that around 50% of participants had some previous knowledge of the pathogen. Therefore, assuming a Type I error of 5% (0.05), and power of 95%, the minimum sample size was calculated as follows:

N = 1.96^2^ × 0.5 × (1 − 0.5)/0.05^2^ = 3.8416 × 0.5 × 0.5/0.0025 = 384 (1)

To post the study invitation, the chief researcher contacted the administrators, requesting preventive authorization to share the link to the questionnaire, including a short description of the aims of the survey. Facebook users who clicked on the invitation text were provided with the full study information, an opportunity to give their informed consent, and a web link to the survey (Google Forms; Google LLC; Menlo Park, California, CA, USA). The survey was conducted in Italian. To be included in the sample, the participant was preventively requested if they was ≥ 18-year-old, and lived in any of the aforementioned communities at the time of the survey. If a potential participant was found not to match the inclusion criteria, the survey closed, and the participant was excluded from the final analyses. The survey was anonymous, and no personal data, such as name, IP address, email address, or personal information unnecessary to the survey, were requested, saved, or tracked. No monetary or other compensation was offered to the participants.

### 2.2. Questionnaire

The questionnaire was formulated in Italian, based on an accurate analysis of similar studies from international literature [27,28,32,34,37,38,39]. Its test–retest reliability was preventively assessed through a survey on 30 subjects, not included in the eventual sampling, completing the questionnaire at two different points in time. All questions were self-reported, and not externally validated. An English translation of the questionnaire is available on request from the corresponding author.

The final questionnaire included the following sections:

#### 2.2.1. Individual Characteristics

Age, Gender, Educational Level, Residence Background (i.e., Urban, i.e., >10,000 Inhabitants; Suburban, i.e., 5000 to 10,000 Inhabitants; Rural, i.e., <5000 Inhabitants) whether They Had: (a) Knowledge of the Terms WNV/WNF; (b) Any Occupational Background in Healthcare Settings

Participants who had any knowledge of WNV/WNF then received the following subsections of the questionnaire, that were otherwise skipped by other responders:

(a) Whether they had any personal interaction (i.e., personal infection, infection in friends, relatives, etc.) with the pathogen and/or the disease.
(b) Whether they were involved in occupational settings that represented an increased risk to be bitten by competent vectors for WNV (i.e., Working outdoors during the greater part of the day; Working with livestock/animals).
(c) Knowledge test: Participants who exhibited any understanding of the terms WNF/WNV received a specifically designed questionnaire on WNV/WNF, including four true/false items based on general characteristics of pathogen, vectors, and disorders [28,35,36,37,38] (i.e., “Cases of WNV have occurred in the Emilia Romagna Region in the last 5 years?”; “People sick with WNF may spread viral infection?”; “Most of the affected people are substantially asymptomatic”; “A vaccine against WNV is to date commercially available?”), and three multiple-choice items (e.g., “How do you think most people get WNV?” … Mosquito bites; “What age group is most likely to get seriously ill with WNV?” … Adults and Elderly (>50 years); “Which behaviors are recommended to avoid getting WNV?”. A General Knowledge Score (GKS) was then calculated as the sum of correctly and incorrectly marked recommendations: When the participants answered correctly, +1 was added to a sum score, whereas a wrong indication or a missing/“don’t know” answer added 0 to the sum score. GKS was then dichotomized by median value in higher vs. lower knowledge status;
(d) Risk perception: Participants were initially asked to rate through a fully labeled 5-points Likert scale the perceived severity of WNV infection (from not severe at all to highly severe; C^WNV^), whether they were worried to get sick with WNV (from not worried at all to highly worried; I^WNV^), and the perceived capability of the respondent to avoid developing WNV infections (from highly unlikely to highly likely; P^WNV^). All factors were then dichotomized in somewhat agree/worried/unlikely vs. somewhat disagree/not worried/likely. As perceived risk has been defined as a function of the perceived probability of an event and its expected consequences [40], a cumulative Risk Perception Score (RPS) was calculated, as follows, and reported a percent value:

RPS = (C^WNF^ × I^WNF^)/P^WNV^ (2)

(e) Vaccine acceptance: Participants were asked whether they would accept vaccination against WNV (yes vs. no), and how much they would be willing to pay (i.e., not interested; free of charge; <10€/shot; 10 to 49€/shot; 50 to 99€/shot; 100 to 149€/shot; 150 to 199€/shot; 200€/shot or more).

#### 2.2.2. Attitudes and Practices

A series of NPIS, including seven PPB (i.e., avoid going outdoors at dawn/dusk; draining standing water from items and the environment around the home; Spreading pesticides around the home and/or in participant’s fields; use of skin repellent before going outdoors; use of skin repellent before working outdoors; wearing long pants, even in summer season; wearing long-sleeved shirts, even in summer season) and five community-level interventions (i.e., tax support of mosquito control programs; fees/penalties for people avoiding mosquito control programs; tax support to help repair damaged screens and dump standing water; fees/penalties for people avoiding repair of damaged screens and dump standing water; community mosquito control program through pesticide spraying) for averting mosquitoes’ bites were presented to all participants, irrespective of their knowledge of WNV/WNF. More precisely, participants were inquired about the frequency with whom they employed the reported PPB using a fully labeled 5-point Likert scale (i.e., never/rarely = 1 time a week, sometimes = 2 to 3 times a week, often = 4 to 5 times a week, always = almost every day), and results were dichotomized as “often” to “always” vs. “never” to “sometimes”. Similarly, attitudes towards community-level interventions were reported using a 5-point Likert scale ranging from “total disagreement” to “total agreement”, and then dichotomized as “somewhat disagree” (i.e., “total disagreement” to “neutral”) vs. “somewhat agree” (i.e., “agreement”, “total agreement”).

### 2.3. Data Analysis

Continuous variables were reported using average ± standard deviations and initially tested for normal distribution (D’Agostino and Pearson omnibus normality test). Correlation between continuous variables was assessed using either Pearson’s correlation test or Spearman’s rank correlation test accordingly to the distribution of the data. Categorical variables were reported as percent values, and their distribution was analyzed through the chi-squared test in respect of three outcome variables:

(a) having or not any previous knowledge of WNV/WNF
(b) being worried about being infected by WNV
(c) supporting community mosquito control programs through pesticide spreading
(d) acceptance of potential WNV vaccine

All categorical variables that at univariate analysis were associated with the aforementioned statuses with a *p* value < 0.20 were included as explanatory variables in a stepwise binary logistic regression analysis model to calculate adjusted odds ratios (aOR) and their respective 95% confidence intervals (95%CI) for the following outcome variables: Being worried about being infected by WNV; supporting community mosquito control programs through pesticide spreading; exhibiting any acceptance of potential WNV vaccine. Statistical analyses were performed using IBM SPSS Statistics 25.0 for Macintosh (IBM Corp., Armonk, NY, USA) and R 4.0.3 (R Core Team (2020), Vienna, Austria) by means of packages epiR (v. 2.0.19), EpiReport (v 1.0.1), fmsb (0.7.0), plot3d (1.3), msm (1.6.8), and sandwich (3.0–0) [41].

### 2.4. Ethical Considerations

Before giving their consent to the survey, participants were briefed that all information would be gathered anonymously and handled confidentially. Participation was voluntary, and the questionnaire was collected only from subjects who had expressed consent for study participation. Identification of individual participants using the presented material is impaired by the lack of personal data, such as the community of residence, the precise occupational setting, etc. Because of the anonymous, observational design, the lack of clinical data about patients, as the study did not configure itself as a clinical trial, a preliminary evaluation by an Ethical Committee was not required, according to the Italian law [42].

## 3. Results

### 3.1. Descriptive Analysis: General Characteristics of the Sample

As shown in Table 1, a total of 469 participants eventually completed the online questionnaire (0.6% of the resident population; 2.1% of the targeted population). Of the respondents, 189 (i.e., 40.3%) were aged 50 years or more, compared to 43.7% (i.e., 36,012 individuals) in the reference communities (*p* = 0.133); 68.4% were females, and 31.6% males. A total of 35.6% reportedly lived with subjects younger than 14 years, and the greater share of respondents resided in suburban settings (i.e., centers having 5000 to 10,000 inhabitants), compared to 19.6% in larger communities (i.e., >10,000 inhabitants), and 17.9% in smaller centers (i.e., <5000 inhabitants), while around a quarter of respondents (25.4%) resided in isolated settlements. Overall, 28.6% of participants reported a university-level of educational achievement, and 11.1% worked in healthcare settings.

Of all respondents, 243 (51.8%) reportedly had any understanding of the term WNV/WNF. As shown in Table 1, this status was more frequently reported among females (54.8%) than in males (45.3%, *p* = 0.054); in subjects aged 40 to 49 years (64.0%) and 30 to 39 years (57%) compared to other age groups (all of them <50%, *p* = 0.003); in respondents living in households with individuals aged 14 years or less (59.3%, *p* = 0.016), and in participants having an educational achievement of University level of higher (72.4% vs. 47.5% in subjects reporting high-school level degree, and 33.7% in participants having a primary school formation achievement). Similarly, the knowledge of the term WNV/WNF was more frequently reported among subjects residing in urban (63.0%) and suburban settings (58.6%), while it was more frequently overlooked by respondents reportedly living in rural (32.1%) or isolated settings (47.1%, *p* < 0.001). Focusing on the occupational factors, the awareness was higher among participants working in healthcare settings (75.0%, *p* < 0.001), and working with animals and cattle (75.0%).

Such subset of respondents was, therefore, inquired about their previous encounters with the pathogen, their understanding of the severity of the disease, and the potential acceptance of a hypothetical immunization against WNV. Eventually, they received the general knowledge test, and the results are summarized in Table 2.

### 3.2. Previous Interactions with WNV/WNF

As shown, 13 out of the 243 participants being aware of the term WNV/WNF (i.e., 5.3%) reported any previous interaction with the pathogen, having received a diagnosis of WNV infection (2.9%), or as it occurred in either a friend (4.5%) or a relative (2.1%).

### 3.3. Assessment of Knowledge about WNV

**Figure 1:**
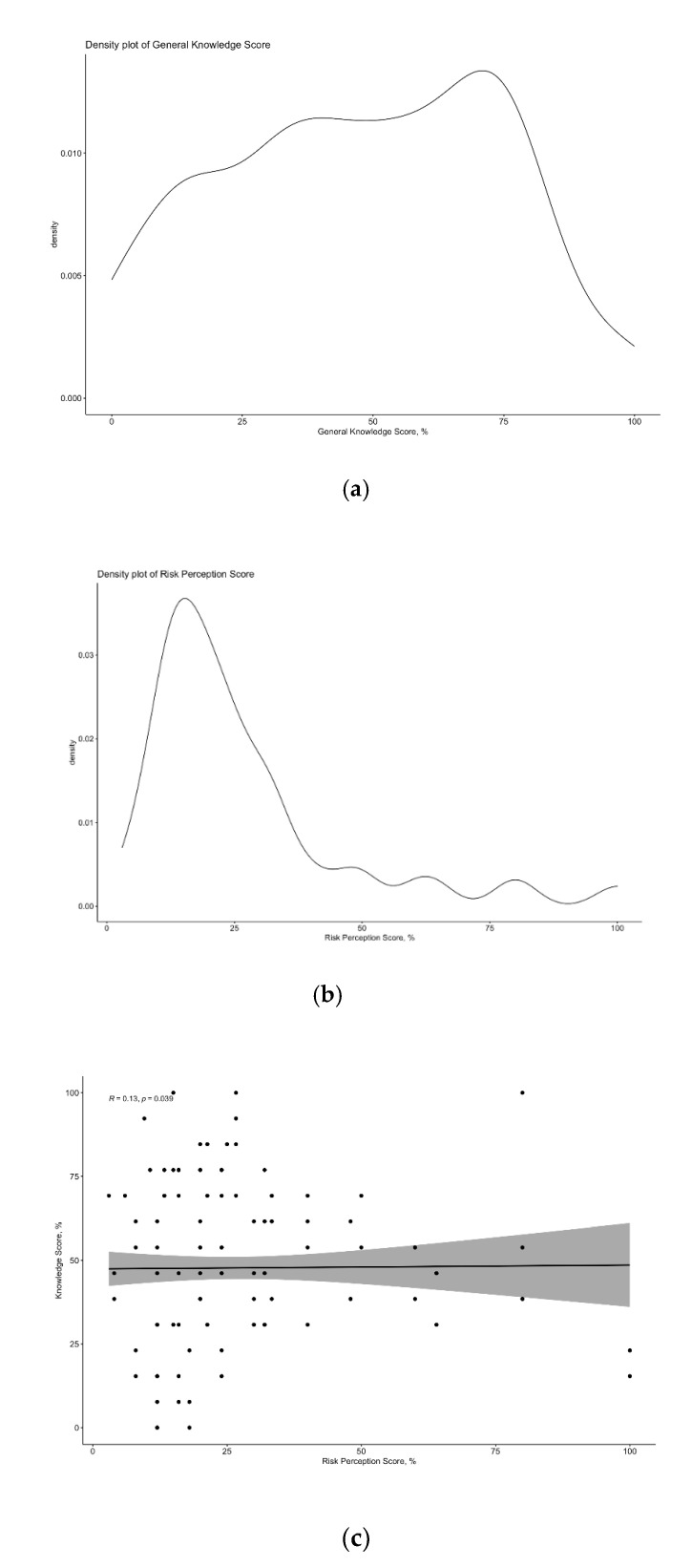
Density plot of General Knowledge Score (**a**) and Risk Perception Score (**b**), showing the skewness of cumulative scores (in both cases, *p* value pf D’Agostino-Pearson’s test < 0.001), and scatter plot for General Knowledge Score vs. Risk Perception Score (**c**), showing the lack of significant correlation between the cumulative scores (Spearman’s rank correlation test *p* = 0.891).

After percent normalization, the mean GKS was relatively low (47.7% ± 26.3; median 40.0%), and its distribution extensively skewed (D’Agostino-Pearson normality test, *p* < 0.001) with very few respondents reporting scores ≥75% (Figure 1). The internal consistency coefficient amounted to Cronbach’s alpha = 0.798, suggesting acceptable reliability of the questionnaire. The majority of respondents (61.3%) were aware that the Emilia Romagna Region was characterized by incident cases of WNV infection, acknowledging WNV infection as transmitted by mosquito bites (84.0%), and not vaccine-preventable (53.9%). On the contrary, participants were affected by uncertainties about the clinical characteristics, including the possible interhuman transmission of the pathogen, that was correctly denied by only 44.4% of respondents, the age group most likely to get seriously ill with WNV infection (i.e., only 32.5% reporting adults and elderly, >50 years old), and features of the infection—more precisely, only 24.3% clearly stated that most of the affected people are substantially asymptomatic.

Further uncertainties were identified when dealing with reported PPB. Behaviors, such as using skin repellent (55.1%), avoiding mosquito-infested areas at dawn and dusk (53.1%), use of mosquito repellents (16.0%), but also wearing long-sleeved shirts (40.7%) and/or long pants (35.4%) even in the summer season, were not diffusely reported. On the contrary, environmental interventions, such as draining or specifically treating standing water from items and the environment around the home, were reported by 87.3% and 61.7% of participants, but only 37.4% of respondents identified as a proper intervention the disinfection against mosquitoes (37.4%).

### 3.4. Risk Perception

The large majority of respondents did characterize WNV infection as a disease of significant severity, but limited occurrence. In fact, 76.5% of them acknowledged WNV infection severity as significant/highly significant: Even though only 13.2% self-styled could protect themselves from the WNV, less than a third of them (31.3%) reported the likeliness of being infected as significant/highly significant. A correspondent RPS equals 26.7% ± 20.35 (D’Agostino-Pearson *p* value < 0.001; Figure 1), was eventually calculated, stressing the relative underestimation of WNV among the sampled residents of the Emilia Romagna Region.

### 3.5. Vaccine Acceptance

A total of 142 respondents (58.4%) exhibited some willingness to receive a potential WNV vaccine. However, when the participants were asked to report how much they were willing to pay for a WNV vaccine, only 1.6% were openly against this intervention. For the majority of respondents (65.4%) the vaccine had to be made available free of charge, or at least should cost less than 50€ by shot (21.8%), with only 8.2% of them accepting a payment up to 99€ by shot.

### 3.6. NPIS and Behaviors 

Focusing on the assessed NPIS, the reporting of PPB by participants was somewhat inconsistent: In fact, only 185 of the total sample (39.4%) identified three or more preventive measures (Table 3), and particularly the drainage of standing water from items and the environment around the home (50.7%), followed by the spread of pesticides in the home environment (i.e., around the home and/or in the fields around it; 33.0%). Even efficient preventive individual behaviors represented by avoiding going outdoors at dawn/dusk (16.8%), using skin repellent before going (42.9%), and working (42.6%) outdoors, but also wearing long pants (26.0%) and long-sleeved shirts (5.5%) in the summer season, were irregularly acknowledged. Interestingly, respondents having any knowledge of WNV/WNF more frequently reported nearly all of the assessed PPB, and particularly avoiding to go outdoors at dawn and dusk (75.9%, *p* < 0.001), followed by wearing long-sleeved shirts even in the summer season (69.2%, *p* = 0.067), draining of standing water (63.9%, *p* < 0.001), using skin repellent before working (63.5%, *p* < 0.001) and going outdoors (59.7%), and the spreading of pesticides (63.9%, *p* < 0.001).

When participants were inquired about their agreement towards community-level measures, the majority of them reported some sort of support to the mosquito control programs, particularly in terms of tax support, both in general (80.2%), for subsidizing the repair of damaged screens and dump standing water (79.3%), and also through the spreading of pesticides (57.2%). On the contrary, the implementation of fees/penalties for either not adhering to the mosquito programs or avoiding appropriate managing of damaged screens and standing water were supported by less than half of respondents (i.e., 41.2% and 46.3%, respectively). Again, respondents having any knowledge of WNV/WNF more frequently acknowledged all the reported interventions, but only for tax exemptions the difference was significant, both for promotion of mosquito control programs, and for interventions aimed to repair damaged screens and dump standing waters (*p* = 0.001 in both cases).

### 3.7. Univariate Analysis

RPS and GKS were not correlated (Spearman’s rank correlation test *p* = 0.891; Figure 1): In other words, a better knowledge status (i.e., fewer misconceptions and/or less personal attitudes guiding the individual behavior) was not associated with a greater risk perception for WNV infection.

In univariate analysis (Table 4), among participants exhibiting any understanding of WNV/WNF, worries on WNV infection were more frequently acknowledged by participants having any previous interaction with the pathogen (69.2%, *p* = 0.046), being less frequently reported by subjects working with animals/cattle (46.7%), of male sex (43.3%, *p* = 0.013), reporting three or more protective behaviors (43.0%, *p* < 0.001), scoring a better knowledge status (42.0%, *p* < 0.001), and perceiving WNV as a severe disease (39.2%, *p* < 0.001). Focusing on the acceptance of the hypothetical WNV vaccine, living with subjects aged 14 years or less was again less frequently reported (49.5%, *p* = 0.019), while the outcome status was more frequently identified among respondents of male gender (82.1%, *p* < 0.001), having a better educational achievement (67.0%, *p* = 0.027), perceiving WNV infection as a severe condition (63.4%, *p* = 0.004), and self-styling could protect from the viral infection (75.0%, *p* = 0.041). Other factors that were more frequently reported among subjects acknowledging the acceptance of a potential WNV vaccine were the reporting three or more protective behaviors (66.9%, *p* = 0.007), being in favor of mosquito control programs performed through the spreading of pesticides (63.2%, *p* = 0.044), and supporting tax exemptions for mosquito control programs (61.7%, *p* = 0.010).

When the sample was assessed as a whole (i.e., 469 participants) on the factors associated with the support of community control programs through pesticide spreading (Table 5), such status was more frequently reported among respondents working with animals/cattle (90.0%, *p* = 0.001), or as an HCW (76.9%, *p* < 0.001), reporting three or more protective behaviors (60.5%, *p* = 0.004), living in suburban/urban areas (57.1%, *p* = 0.015), and more in general among participants promoting mosquito control programs—either through fees and penalties for people avoiding their contribution to the mosquito control programs (69.9%, *p* < 0.001) or not intervening on damaged screens and standing water (66.8%, *p* < 0.001), or by promoting tax exemptions, both in general (60.1%, *p* < 0.001), and aimed to repair damaged screens and dump standing water (58.1%, *p* = 0.001). On the contrary, the aforementioned status was less frequently identified in older age groups (35.1% vs. 46.0%, *p* = 0.016).

### 3.8. Multivariate Analysis 

In regression analysis (Table 6), the outcome variable represented by being worried by WNV infection was associated with the explanatory variables represented by living in a household with subjects aged 14 years or less (aOR 3.624; 95%CI 1.712 to 7.674), reporting a better GKS (aOR 3.852, 95%CI 1.664 to 8.918), perceiving WNV infection as a severe condition (aOR 20.288, 95%CI 5.083 to 80.972), and being favorable to mosquito control programs through pesticide spreading (aOR 5.098, 95%CI 2.207 to 11.773).

In turn, acceptance of potential WNV vaccines was negatively associated with living in a household with subjects aged 14 years or less (aOR 0.461, 95%CI 0.246 to 0.861), and positively associated with being of male gender (aOR 5.251, 95%CI 2.452 to 11.246), as well as supporting tax exemptions for mosquito control programs (aOR 2.716, 95%CI 1.087 to 6.783).

Eventually, supporting mosquito control programs through pesticide spreading was negatively associated with the explanatory variable represented by the older age group (aOR 0.569, 95%CI 0.362 to 0.893), and positively associated with working as HCW (aOR 2.326, 95%CI 1.100 to 4.920), working with animal/livestock (aOR 13.948, 95%CI 2.793 to 69.653), and being favorable to supporting mosquito control programs using tax exemptions (aOR 4.069, 95%CI 2.098 to 7.893).

## 4. Discussion

The last decade was characterized by a sustained circulation of WNV in Northern Italy [9,15,43]. However, even in residents from areas characterized by a high or very high incidence of WNV infections (i.e., Emilia Romagna Region), nearly half of respondents had not heard of WNV/WNF, and among those who did, most of them exhibited significant uncertainties in terms of understanding of WNV-related issues, including the actual risk perception. Although a large share of respondents showed some awareness of WNV transmission, acknowledging WNV infections as a serious issue that is very unlikely to avoid in the years to come, most of them reported little or no concern about getting it. Moreover, when focusing on the adherence to NPIS, a significant share of respondents was somewhat reluctant to cope with appropriate PPB or in promoting community-level preventive measures.

In our study, we specifically inquired three outcomes, represented by being worried about WNV infection, being favorable to community-level interventions against mosquitoes using pesticides, and the eventual acceptance of a hypothetical human vaccine against WNV. Three distinctive patterns were therefore identified, but some shared characteristics should be highlighted.

First and foremost, knowledge status only had a predictive value for worries towards WNV infection, as well as acknowledging the potential severity of the resulting disease. In fact, such remark conflicts with the extensive agreement that an improved understanding of WNV/WNF significance may improve the adherence and the promotion of appropriate attitudes and practices [38,44]. Similarly, other factors usually associated with a better acceptance of appropriate preventive interventions—including vaccinations, had mixed issues. For example, living in a household that includes subjects younger than 14-year-old was positively associated with increased worries towards getting the WNV infection, while it was a negative predictor towards the acceptance of the vaccine. In turn, and with an appreciable contrast to previous reports, neither a better knowledge status nor the acknowledgment of the actual consequences of WNV infection were effective predictors of vaccine acceptance [33,37]. While in some previous reports, the excess of confidence and lack of concern were partially explained through the diffuse reporting of collective and/or personal protective practices [37], our sample was extensively unsatisfying in terms of acceptance of NPIS and PPB, and particularly those characterized by a minimal impact on individual attitudes and practices, such as avoiding the potential and unnecessary individual exposures to mosquito-infested areas [27,32,34,37,38].

Interestingly, people exhibiting some acceptance for community-level interventions, and more precisely the pesticide spreading, were more likely to be working as HCWs or involved in the managing of livestock and/or other animals, presumptively because a better understanding of the health risks associated with pests, including the vectors for WNV. The acceptance of pesticide treatment was somewhat unexpected, firstly because of its overall extent (52.2% of all participants were somewhat favorable to these interventions), but also for the diffuse agreement among younger participants [37]. However, it should be stressed that some previous reports have questioned the otherwise seemingly obvious restraints of younger age groups towards pesticides [45,46].

Albeit the aforementioned remarks were somewhat inconsistent with available studies, most of these findings were not unexpected [27,28,38,39]. According to the Health Belief Model, a person’s belief in a health threat, as well as the belief in the effectiveness of the recommended health behavior or action, represent the main predictors for the likelihood that people will adopt the behavior [37]. WNV usually causes a mild disorder, often described as “summer flu”, that in turn is quite similar to other arboviral infections [47], while only a reduced share of total cases develops the more severe WNND [5,15,19,21]. In other words, the actual severity of WNV infections may be radically overlooked, and the trade-off between appropriate NPIS and the risk for WNV infection may be perceived by the general population as disproportionately unbalanced [27,32,37,38].

Even though a previous study from Tuiten et al. [38] suggested a direct relation between perceptions on WNV and acceptance of NPIS, these results are not consistently reported in available studies [32,37]. Our research also suggests a more complex correlation between residents’ knowledge and risk perception. On the one hand, while knowledge status and risk perception were unrelated, a better understanding of WNV/WNF was an effective predictor for being worried about WNV infection. On the other hand, previous reports have stressed that even in subjects properly aware of the potential severity of WNV infections, a significant share of the general population exhibits little or no concern about getting the pathogen and developing the disease [37]. This attitude may be explained by inappropriate confidence in PPB, and more in general in NPIS, but also in the often-missed link between competent mosquito vector and the WNV. Furthermore, in our sample, a considerable share of respondents (around 16%) who had some knowledge of WNV were unable to identify the mosquitoes as the vector for this pathogen, while the large majority of them improperly identified WNV infection as an inter-human communicable disease.

In other words, when dealing with WNV/WNF it should be kept in mind that, without an inter-human transmission of the pathogen, all NPIS target an often-overlooked vector, i.e., the mosquitoes, being in turn improperly disregarded. Even though Northern Italy, as well as other European areas, have been historically plagued by other mosquito-borne pathogens, such as malaria, since the end of World War II and the subsequent interventions through swamp reclamations and pesticide dispersal, mosquitoes have been largely regarded as nothing more than a seasonal, annoying, and even irritant, bugs, but substantially indolent [48]. Consequently, the participants may be improperly optimist when pondering their chances to avoid WNV infections, while some PPB, such as applying insect skin repellents, may be improperly applied because of their potential side effects (i.e., skin sensitization to the chemicals), or simply not applied [32,37]. For example, in their survey, Pogreba-Brown et al. reported that approximately one in eight people did not cope with basic mosquito reduction strategies of emptying standing water and reducing potential habits, despite respondents cited them as the most effective factors in reducing mosquitoes [34].

Interestingly, even though the majority of respondents were aware that no effective human WNV vaccination is to date available, a significant share of them was willing to invest their money in a WNV vaccine, presumptively perceiving a benefit in protecting themselves from WNV. In previous reports, such attitude was either associated with a better knowledge status [37], or with an occupational background in healthcare settings [33], while in our survey not only knowledge status, but also perceiving WNV as a potential health threat, and acknowledging the potential risk to get the pathogen, were identified as significant effectors. In this regard, while Mitchell et al. have previously hinted towards a mutually exclusive status between a more favorable attitude towards WNV vaccine and acceptance of NPIS [37], in our survey, acceptance of a potential vaccine was associated with a better agreement for tax exemptions for interventions contributing to mosquito control programs. In other words, the personal cost-benefit analysis that includes the acceptance of NPIS may be even more complicated than previously assumed, possibly mirroring the different background from North American studies [27,32,37,38,39].

*Limitations*. Despite the potential interest, our study has some significant limitations. To begin with, it shares the implicit limits of all Internet-based surveys [6,49,50]. Albeit reliable, cost-effective, and mostly much faster than a paper-based survey, internet-based surveys are usually affected by the “self-selection” participants. As a consequence, the final sample could over-represent certain sub-groups, and particular subjects that—because of their better literacy or younger age are more accustomed to sharing personal information through internet access, but also individuals exhibiting a proactive attitude or greater knowledge about the assessed topic. In the same way, not participating could be understood as a negative attitude or a lack of knowledge about vaccinations. In this regard, our sample was certainly affected by some degree of self-selection, as suggested by the over-representation of subjects of the female gender, but the representation of the various age groups was satisfyingly consistent with the reference population.

Second, even though some of the assessed items, and notably those dealing with the use of pesticides, were associated with results that are significantly against a “socially appropriated” behavior, it is reasonable that some of the items assessed through the knowledge test may be affected by a significant social desirability bias. Therefore, our results could have ultimately overstated the share of individuals having an effective understanding of WNV/WNF associated issues, including the very same knowledge of this disorder [51,52,53].

Third, although the number of participants fulfilling the online questionnaire exceeded the preventive estimate from sample size calculation, our study focused on a small area of the Po River Valley, and included less than 1% of the resident population from the targeted municipalities. In other words, our results should be cautiously interpreted as representative not only of the national level, but also of the Emilia Romagna Region as a whole.

Fourth, while a certain selection is usually performed by social media managers of specific discussion groups (e.g., by registering only subjects who receive a specific invitation by the manager; answering specific “selection” questions; etc.), we cannot rule out that some of the respondents were residents outside the targeted municipalities, not fulfilling our initial selection criteria, and compromising the actual representativity of the sample.

## 5. Conclusions

Our study suggests that residents from the high-risk area of the Emilia Region exhibit an extensive lack of knowledge on WNV/WNF, with inappropriate risk awareness. Moreover, adherence and acceptance of NPIS were largely unsatisfying, as our results suggest that a significant share of sampled participants ignores, or only partially applies, official recommendations to avoid not only WNV/WNF, but also other mosquito-borne diseases.

Unfortunately, as knowledge status was not unequivocally associated with more appropriate risk perception, being otherwise identified as a significant predictor of worries towards WNV infection, it is plausible that filling such information gaps might improve the rate of proper NPIS and the acceptance of community-level interventions. Moreover, the potential acceptance of forthcoming human WNV vaccines may benefit from such intervention.

As WNV infection may be effectively countered through effective behavioral practices, improving this way the prevention of all mosquito-borne infections among the general population of high-risk areas, and increasing their health literacy could be, therefore, instrumental and cost-effective in reducing the potential spreading of WNV infections.

## Figures and Tables

**Table 1:** General characteristics of 469 residents from the Provinces of Parma and Reggio Emilia participating in the survey (2021), broken down by their understanding of the term West Nile Virus (WNV)/West Nile Fever (WNF).

Characteristics	TOTAL	Knowledge of the Term “West Nile Virus”/“West Nile Fever”	
	No./469, %		
		Yes (No., %)	*p* Value *
Gender			0.054
Male	148, 31.6%	67, 45.3%	
Female	321, 68.4%	176, 54.8%	
Age, years			0.003
20–29	51, 10.9%	23, 45.1%	
30–39	93, 19.8%	53, 57.0%	
40–49	136, 29.0%	87, 64.0%	
50–59	125, 26.7%	56, 44.8%	
60 and more	64, 13.6%	25, 39.1%	
Education			<0.001
Primary School	95, 20.3%	32, 33.7%	
High school	240, 51.2%	114, 47.5%	
University or higher	134, 28.6%	97, 72.4%	
Household with subjects aged 14 years or less	167, 35.6%	99, 59.3%	0.016
Residence location			<0.001
Urban (i.e., >10,000 inhabitants)	92, 19.6%	58, 63.0%	
Suburban (i.e., 5000 to 10,000 inhabitants)	174, 37.1%	102, 58.6%	
Rural (i.e., <5000 inhabitants)	84, 17.9%	27, 32.1%	
Isolated	119, 25.4%	56, 47.1%	
Working in healthcare settings	52, 11.1%	39, 75.0%	<0.001
Working outdoors during the greater part of the day	31, 6.6%	17, 54.8%	0.727
Working with animals/cattle	20, 4.3%	15, 75.0%	0.034

* chi-squared test.

**Table 2:** Attitudes, practices, and knowledge status on West Nile Virus (WNV) and West Nile Fever (WNF) in 243 residents from the provinces of Parma and Reggio Emilia participating in the survey and having any self-reported knowledge of the WNV.

Attitudes and Practices	No./243, %
Previous diagnosis of WNV?	7, 2.9%
Previous diagnosis of WNV in relatives?	5, 2.1%
Previous diagnosis of WNV in friends?	11, 4.5%
Any previous interaction with WNV	13, 5.3%
Do you believe WNV may cause a serious disease?	
Somewhat agree	186, 76.5%
Somewhat disagree	57, 23.5%
How worried are you that you will get sick with WNV?	
Somewhat worried	76, 31.3%
Somewhat not worried	167, 68.7%
How likely is it that you will be able to protect yourself from WNV?	
Somewhat likely	32, 13.2%
Somewhat unlikely	211, 86.8%
Risk Perception Score (average ± S.D.)	26.7% ± 20.3
Willing to accept a WNV vaccine (if available)?	
Yes	142, 58.4%
No	101, 41.6%
Amount willing to pay for WNV vaccine (if available)	
Not interested/Against vaccination	4, 1.6%
WNV vaccine should be available free of charge	159, 65.4%
<10€/shot	18, 7.4%
10–49€/shot	35, 14.4%
50–99€/shot	20, 8.2%
100–149€/shot	4, 1.6%
150–199€/shot	3, 1.2%
200€/shot or more	0, -
**Knowledge test**	
1. Cases of WNV have occurred in the Emilia Romagna Region in the last 5 years?	
True (CORRECT)	149, 61.3%
False/Don’t know	94, 35.2%
2. How do you think most people get WNV?	
Eating or drinking contaminated food or water	0, -
Mosquito bites (CORRECT)	204, 84.0%
Tick bites	2, 0.8%
Contact with Birds	0, -
Contact with cattle	2, 0.8%
Contact with sick people	0, -
Don’t know	35, 14.4%
3. What age group is most likely to get seriously ill with WNV?	
Young children (<11 years)	5, 2.1%
Adolescents (11–18 years)	0, -
Young adults (19–25 years)	0, -
Adults (26–50 years)	6, 2.5%
Adults and Elderly (>50 years) (CORRECT)	79, 32.5%
All of the above	49, 20.2%
Don’t know	104, 42.8%
4. People sick with WNF may spread viral infection?	
True	29, 11.9%
False (CORRECT)	108, 44.4%
Don’t know	106, 43.6%
5. Most of the affected people are substantially asymptomatic	
True (TRUE)	59, 24.3%
False	71, 29.2%
Don’t know	113, 46.5%
6. A vaccine against WNV is to date commercially available?	
True	6, 2.5%
False (FALSE)	131, 53.9%
Don’t know	106, 43.6%
7. Which behaviors are recommended to avoid getting WNV?	
Drain standing water from items and the environment around the home (CORRECT)	200, 87.3%
Treat standing water from items and the environment around the home (CORRECT)	150, 61.7%
Preventive vaccination	22, 9.1%
Wearing long-sleeved shirts (even in summer season) (CORRECT)	99, 40.7%
Wearing long pants (even in summer season) (CORRECT)	86, 35.4%
Use of skin repellent (CORRECT)	134, 55.1%
Disinfection against mosquitos (CORRECT)	91, 37.4%
Avoid going in mosquito-infested areas at dawn and dusk (CORRECT)	129, 53.1%
Use of mosquito repellents (such as lemongrass candles, etc.) (CORRECT)	39, 16.0%
Put the end of your pants inside of your shoes	20, 8.2%
Use of large hats	7, 2.9%
Knowledge Score (average ± S.D.)	47.7% ± 26.3
Knowledge Score > median (46.2%)	119, 49.0%

Note. WNV = West Nile Virus; WNF = West Nile Fever.

**Table 3:** Common Non-Pharmaceutical Interventions (NPIS) against West Nile Virus (WNV)/West Nile Fever (WNF) in terms of personal protective behaviors (reported by participants as employed “often” and “always”) and community-level interventions (reported by participants as “agreed” and “totally agreed”).

Reported Non-Pharmaceutical Intervention	TOTAL	Knowledge of the Terms	
		“West Nile Virus”/“West Nile Fever”	
	No./469,%	Yes (No./243, %)	*p* Value *
Personal Protective Behaviors			
(Often/Always)			
Three or more personal protective behaviors	185, 39.4%	121, 65.4%	<0.001
Avoid going outdoors at dawn and dusk	79, 16.8%	60, 75.9%	<0.001
Use of skin repellent before going outdoors	201, 42.9%	120, 59.7%	0.003
Use of skin repellent before working outdoors	200, 42.6%	127, 63.5%	<0.001
Wearing long-sleeved shirts (even in summer season)	26, 5.5%	18, 69.2%	0.067
Wearing long pants (even in summer season)	122, 26.0%	56, 45.9%	0.129
Drain standing water from items and the environment around the home	238, 50.7%	152, 63.9%	<0.001
Spreading pesticides around the homes/in your fields	144, 33.0%	86, 59.7%	0.264
Community-level interventions			
(Agreement/Total Agreement)			
Tax support of mosquito control programs	376, 80.2%	209, 55.6%	0.001
Fees/penalties for people avoiding mosquito control programs.	193, 41.2%	110, 57.0%	0.060
Tax support to help repair damaged screens and dump standing water.	372, 79.3%	207, 55.6%	0.001
Fees/penalties for people avoiding repair of damaged screens and dump standing water.	217, 46.3%	116, 53.5%	0.509
Support of community mosquito control program through pesticide spreading.	245, 57.2%	134, 54.7%	0.192

* chi-squared test.

**Table 4:** Association of the status being worried about being infected by West Nile Virus (WNV), acceptance of potential WNV vaccines, and characteristics of the study participants in the subgroup of respondents having any knowledge of WNV. Univariate analysis was performed using chi-squared test.

Variable	TOTAL	Worried about being Infected by WNV		Acceptance of Potential WNV Vaccine	
	No./243,%	Yes	*p* Value *	Yes	*p* Value *
		(No.,%)		(No.,%)	
Male sex	67, 27.6%	29, 43.3%	0.013	55, 82.1%	<0.001
Age > 50 years	81, 33.3%	29, 35.8%	0.353	44, 54.3%	0.357
University degree	114, 46.9%	31, 27.2%	0.188	65, 67.0%	0.027
Household with subjects aged 14 years or less	99, 40.7%	38, 38.4%	0.048	49, 49.5%	0.019
Living in suburban/urban area	160, 65.8%	50, 31.3%	0.990	96, 60.0%	0.583
Working as HCW	39, 16.0%	13, 33.3%	0.762	25, 64.1%	0.433
Worried about being infected by WNV	76, 31.3%	-	-	50, 65.8%	0.117
Any previous interaction with WNV	13, 5.3%	9, 69.2%	0.046	11, 64.7%	0.773
Knowledge Score > median (46.2%)	119, 49.0%	50, 42.0%	<0.001	67, 56.3%	0.595
Perceiving WNV as a severe disease	186, 76.5%	73, 39.2%	<0.001	118, 63.4%	0.004
Perceiving able to protect from WNV	32, 13.2%	10, 31.3%	0.997	24, 75.0%	0.041
Reporting three or more protective behaviors	121, 49.8%	52, 43.0%	<0.001	81, 66.9%	0.007
Working outdoors during the greater part of the day	17, 7.0%	6, 35.3%	0.711	9, 52.9%	0.634
Working with animals/cattle	15, 6.2%	7, 46.7%	0.184	10, 66.7%	0.504
Favorable to tax support of mosquito control programs	209, 86.0%	62, 29.7%	0.179	129, 61.7%	0.010
Favorable to fees/penalties for people avoiding mosquito control programs	110, 45.3%	33, 30.0%	0.696	66, 60.0%	0.653
Favorable to tax support to help repair damaged screens and dump standing water	207, 85.2%	67, 32.4%	0.379	120, 58.0%	0.724
Favorable to fees/penalties for people avoiding repair of damaged screens and dump standing water	116, 47.7%	34, 29.3%	0.528	70, 60.3%	0.564
Favorable to tax support of mosquito control programs through pesticides	134, 55.1%	48, 35.8%	0.090	86, 64.2%	0.044

Note. HCW = healthcare workers. * = chi-squared test.

**Table 5:** Association of the status of supporting community mosquito control programs through pesticide spreading with individual characteristics of the study participants. Univariate analysis was performed using chi-squared test.

Variable	Total	Support Community Mosquito Control Program through Pesticide Spreading	
	(No./469, %)	Yes (No., %)	*p* Value *
Knowledge of the terms “West Nile Virus”/“West Nile Fever”	243, 51.8%	134, 55.1%	0.192
Male sex	148, 31.6%	76, 51.4%	0.794
Age > 50 years	189, 40.3%	86, 45.5%	0.016
University degree	134, 28.6%	79, 59.0%	0.066
Household with subjects aged 14 years or less	167, 35.6%	89, 53.3%	0.734
Living in suburban/urban area	266, 56.7%	152, 57.1%	0.015
Working as HCW	52, 11.1%	40, 76.9%	<0.001
Reporting three or more protective behaviors	185, 39.4%	112, 60.5%	0.004
Working outdoors during the greater part of the day	31, 6.6%	15, 48.4%	0.657
Working with animals/cattle	20, 4.3%	18, 90.0%	0.001
Favorable to tax support of mosquito control programs	376, 80.2%	226, 60.1%	<0.001
Favorable to fees/penalties for people avoiding mosquito control programs	193, 41.2%	135, 69.9%	<0.001
Favorable to tax support to help repair damaged screens and dump standing water	372, 79.3%	216, 58.1%	0.001
Favorable to fees/penalties for people avoiding repair of damaged screens and dump standing water	217, 46.3%	145, 66.8%	<0.001

Note. HCW = healthcare workers; * = chi-squared test.

**Table 6:** Multivariate analysis of factors associated with the outcome variables represented by being worried about being infected by West Nile Virus (WNV), accepting a potential WNV vaccine, and supporting mosquito control programs through pesticide spreading. The assessed model included all factors that in univariate analysis were associated with the outcome variables with a chi-squared *p* value < 0.20. Adjusted Odds Ratios (aOR) with their correspondent 95% confidence intervals (95%CI) were calculated using binary logistic regression analysis.

Variable	Worried about being Infected by WNV		Acceptance of Potential WNV Vaccine		Support Community Mosquito Control Programs through Pesticide Spreading	
	aOR	95%CI	aOR	95%CI	aOR	95%CI
Knowledge of the terms “West Nile Virus”/“West Nile Fever”	-	-	-	-	0.768	0.491; 1.201
Male sex	1.348	0.649; 2.801	5.251	2.452; 11.246	-	-
Age > 50 years	-	-	-	-	0.569	0.362; 0.893
University degree	0.405	0.185; 0.886	1.351	0.720; 2.533	0.859	0.517; 1.428
Household with subjects aged 14 years or less	3.624	1.712; 7.674	0.461	0.246; 0.861	-	-
Living in suburban/urban area	-	-	-	-	1.458	0.952; 2.233
Working as HCW	-	-	-	-	2.362	1.100; 1.906
Worried about being infected by WNV	-	-	1.472	0.725; 2.989	-	-
Any previous interaction with WNV	1.887	0.555; 6.415	-	-	-	-
Knowledge Score > median (46.2%)	3.852	1.664; 8.918	-	-	-	-
Perceiving WNV as a severe disease	20.228	5.083; 80.972	0.888	0.396; 1.988	-	-
Perceiving able to protect from WNV	-	-	2.276	0.916; 5.652	-	-
Reporting three or more protective behaviors	1.696	0.814; 3.532	1.647	0.882; 3.074	1.222	0.783; 1.906
Working with animals/cattle	1.696	0.814; 3.532	-	-	13.948	2.793; 69.653
Favorable to tax support of mosquito control programs	0.129	0.042; 0.399	2.716	1.087; 6.783	4.069	2.098; 7.893
Favorable to fees/penalties for people avoiding mosquito control programs	-	-	-	-	1.585	0.854; 2.941
Favorable to tax support to help repair damaged screens and dump standing water	-	-	-	-	1.566	0.8709; 2.818
Favorable to fees/penalties for people avoiding repair of damaged screens and dump standing water	-	-	-	-	1.748	0.951; 3.216
Favorable to tax support of mosquito control programs through pesticides	5.098	2.207; 11.773	0.999	0.529; 1.889	-	-

Note. HCW = healthcare workers.

## Data Availability

The data presented in this study are available on request from the corresponding author.

## Appendix A

**Table A1:** Characteristics of the municipalities involved in the survey.

Municipality	Population (Census 2020)	Share of the Population Aged 50-Years Old or Older	Participants to Facebook Local Group(s) (No./Population,%)	Surface (Km^2^)
(Province)				
San Secondo Parmense	5758	43.8%	1057, 18.4%	37.71
(Parma)				
Sissa Trecasali	7788	45.1%	807, 10.4%	72.72
(Parma)				
Colorno	9103	41.7%	1285, 14.1%	48.41
(Parma)				
Torrile	7695	39.3%	4625, 60.1%	37.15
(Parma)				
Sorbolo Mezzani	12,602	44.5%	2253, 17.8%	66.98
(Parma)				
Brescello	5656	41.4%	2280, 40.3%	24.04
(Reggio Emilia)				
Poviglio	7167	44.2%	2053, 28.6%	43.55
(Reggio Emilia)				
Boretto	5314	43.4%	2648, 49.8%	18.11
(Reggio Emilia)				
Gualtieri	6338	37.7%	1874, 29.6%	35.65
(Reggio Emilia)				
Guastalla	14,896	45.8%	3202, 21.5%	52.93
(Reggio Emilia)				
Total	82,317	43.7%	22,085, 26.8%	437.25
